# Secondary Hypertension and Renal Atrophy Unmasking Takayasu Arteritis With Renal Artery Stenosis

**DOI:** 10.7759/cureus.110793

**Published:** 2026-06-13

**Authors:** Laktib Nabil, Selma Saidi, Yahya Sqalli Houssaini, Zouhair Lakhal, Aatif Benyass

**Affiliations:** 1 Cardiology Center, Mohammed V Military Teaching Hospital, Mohammed V University, Rabat, MAR; 2 Cardiology, Hôpital Universitaire Avicennes, Rabat, MAR; 3 Radiology, Mohammed V Military Teaching Hospital, Mohammed V University, Rabat, MAR

**Keywords:** large vessel vasculitis, renal atrophy, renovascular hypertension, secondary hypertension, takayasu arteritis

## Abstract

Takayasu arteritis is a rare inflammatory arteriopathy predominantly affecting young women and representing an important, often under-recognized, cause of secondary hypertension. Diagnosis is frequently delayed due to nonspecific early manifestations, while advanced vascular lesions may already be established at presentation.

A 25-year-old woman with no prior history was referred for incidentally discovered hypertension. Workup excluded common secondary causes but showed elevated inflammatory markers. Imaging revealed severe left renal atrophy with renal artery occlusion, circumferential suprarenal abdominal aortic wall thickening, and superior mesenteric artery ostial stenosis on computed tomography angiography. Takayasu arteritis was diagnosed. Hypertension was attributed to renovascular disease, but revascularization was not indicated due to advanced renal atrophy. The patient was treated with corticosteroids and optimized antihypertensive therapy.

This case underscores the insidious presentation of Takayasu arteritis and the pivotal role of imaging in diagnosing active inflammatory lesions. It highlights renovascular hypertension as a key manifestation and emphasizes the importance of assessing renal viability when considering revascularization. In advanced unilateral renal atrophy, the benefit of intervention is limited, and management should prioritize medical therapy.

Takayasu arteritis should be considered in young patients with unexplained hypertension and inflammatory syndrome. Early recognition and imaging are essential to guide management and avoid unnecessary interventions in advanced disease.

## Introduction

Takayasu arteritis is a rare chronic granulomatous large-vessel vasculitis affecting the aorta and its major branches, with an estimated incidence of one to three cases per million annually, predominantly in young women [1]. Progressive arterial inflammation leads to vascular wall thickening, stenosis, occlusion, or aneurysm formation. Although uncommon, it is an important cause of secondary hypertension in young patients, often diagnosed late because of nonspecific systemic manifestations and insidious vascular progression [2]. Renal artery involvement commonly induces renovascular hypertension through activation of the renin-angiotensin-aldosterone system secondary to reduced renal perfusion. Complete unilateral renal artery occlusion leading to severe renal atrophy at presentation remains uncommon and reflects advanced, structurally destructive disease [3].

We report the case of a 25-year-old woman with incidentally discovered severe secondary hypertension associated with systemic inflammatory markers and imaging evidence of diffuse large-vessel involvement, including suprarenal aortic wall thickening, superior mesenteric artery stenosis, and complete left renal artery occlusion with renal atrophy. This constellation highlights several key clinical and diagnostic challenges: the often silent evolution of large-vessel vasculitis, the pivotal role of cross-sectional imaging in defining both inflammatory and chronic fibrotic lesions, and the mechanisms of renovascular hypertension in large-vessel vasculitis. Finally, the case underscores therapeutic decision-making dilemmas, especially the limited role of revascularization in chronically occluded, non-salvageable renal units, and the importance of prioritizing optimal medical immunosuppressive therapy and blood pressure control.

## Case presentation

A 25-year-old woman with no significant past medical history was referred for evaluation of newly diagnosed hypertension, incidentally detected during a routine consultation, without associated constitutional symptoms.

On admission, blood pressure was 168/102 mmHg, with a heart rate of 82 beats per minute, respiratory rate of 16 breaths per minute, temperature of 36.8 °C, and oxygen saturation of 98% on room air. Physical examination was unremarkable, with symmetric peripheral pulses, no inter-arm blood pressure difference, and no vascular bruits. There was no peripheral edema, and the abdominal examination was normal.

Initial laboratory evaluation (Table 1), including renal function and electrolytes, was within normal limits. Secondary hypertension workup, including thyroid-stimulating hormone, plasma, and urinary metanephrines, was unremarkable. Urinalysis showed no proteinuria or hematuria. Plasma aldosterone concentration was 50 ng/dL (upright reference range: 7-30 ng/dL) and plasma renin activity was increased at 10 ng/mL/h (upright reference range: 0.5-4 ng/mL/h). Inflammatory markers were also elevated, with C-reactive protein at 18 mg/L (reference <5 mg/L) and erythrocyte sedimentation rate at 42 mm/h (reference <20 mm/h), suggesting an inflammatory process. D-dimer level was 300 ng/mL (reference range: <500 ng/mL). Autoimmune serologies, including antinuclear antibodies and antineutrophil cytoplasmic antibodies, were negative.

**Table 1:** Laboratory findings

Laboratory tests	Patient’s findings	Normal range
Sodium (mmol/L)	140	135-145
Potassium (mmol/L)	4	3.5-5.1
Serum bicarbonate (mmol/L)	23	22-24
Urea (mmol/L)	4	2.5-7.5
Creatinine (µmol/L )	50	44-97
C-reactive protein (mg/L)	18	<5
Erythrocyte sedimentation rate (mm/h)	42	<20-30
Plasma renin activity (ng/mL/h)	10	0.5-4
Aldosterone (ng/dL)	50	7-30
Thyroid-stimulating hormone (mIU/L)	2.5	0.4-4.0
Plasmatic Metanephrine (nmol/L)	0.3	<0.50
Normetanephrine (nmol/L)	0.5	<0.90
Urinary metanephrines (µg/24 h)	200	<400
D-dimer (ng/mL)	300	<500

**Figure 1:**
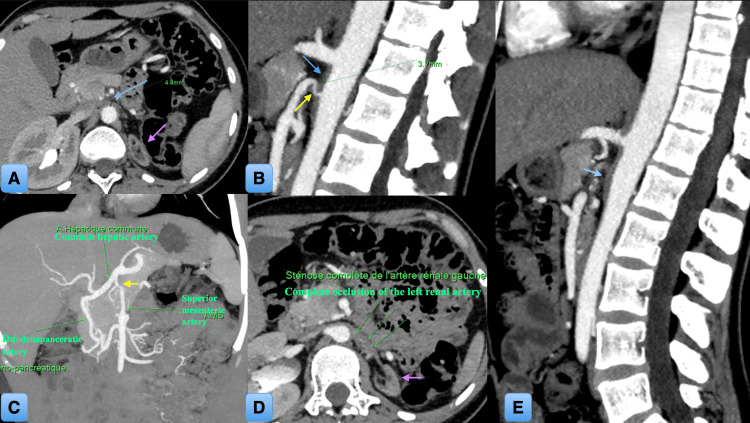
Computed tomography angiography findings consistent with large-vessel vasculitis involving the abdominal aorta and its major branches Panel A: Coronal reconstruction demonstrating marked renal asymmetry with severe left renal atrophy (pink arrow), the left kidney measuring 53 mm in bipolar length, indicating chronic hypoperfusion and irreversible ischemic damage. Associated circumferential mural thickening of the suprarenal abdominal aorta (blue arrow) is suggestive of active large-vessel vasculitis. Panel B: Axial image showing concentric inflammatory thickening of the aortic wall (blue arrow) together with severe ostial stenosis of the superior mesenteric artery (yellow arrow), reflecting diffuse inflammatory involvement of major abdominal vessels. Panel C: Sagittal reconstruction illustrating critical ostial stenosis of the superior mesenteric artery (yellow arrow) with preserved distal perfusion through enlarged pancreaticoduodenal collateral vessels, indicating chronic progressive mesenteric ischemia with compensatory collateralization. Panel D: Coronal angiographic image revealing complete occlusion of the left renal artery from its ostium (green arrows), associated with marked ipsilateral renal atrophy (pink arrow), consistent with advanced renovascular disease secondary to Takayasu arteritis. Panel E: Axial image demonstrating circumferential thickening of the suprarenal abdominal aortic wall (blue arrow), consistent with inflammatory aortic involvement in Takayasu arteritis.

Abdominopelvic computed tomography revealed marked renal asymmetry with a severely atrophic left kidney measuring 53 mm in bipolar length compared with a 115 mm right kidney, without lithiasis or urinary obstruction. Computed tomography angiography (Figure 1) demonstrated outward, concentric and circumferential suprarenal abdominal aortic wall thickening, severe ostial stenosis of the superior mesenteric artery with collateral opacification, and complete occlusion of the left renal artery from its origin. No imaging evidence of superimposed intraluminal aortic thrombus was identified. Delayed contrast excretion was noted in the left kidney, with preserved corticomedullary differentiation. The right kidney showed compensatory hypertrophy with preserved function.

In the context of a young patient with systemic inflammation and characteristic large-vessel involvement, a diagnosis of Takayasu arteritis was established. Hypertension was attributed to renovascular disease secondary to left renal artery occlusion.

Treatment was initiated with systemic corticosteroids (prednisone 1 mg/kg/day), low-dose aspirin 75 mg daily, and antihypertensive therapy with amlodipine 5 mg daily and valsartan 80 mg daily, resulting in progressive blood pressure control.

Given complete left renal artery occlusion, severe renal atrophy, and lack of functional renal salvageability, no indication for revascularization was retained. The patient was scheduled for close clinical, biological, and imaging follow-up to monitor disease activity and vascular progression.

After one month, the patient was clinically well with controlled blood pressure at 120/70 mmHg. Inflammatory markers had normalized, and she remained asymptomatic under ongoing corticosteroid and antihypertensive therapy.

## Discussion

Takayasu arteritis is a chronic, granulomatous large-vessel vasculitis involving the aorta and its major branches, predominantly affecting young women and representing an important cause of secondary hypertension in this population. 

The clinical presentation is classically biphasic, comprising an early systemic inflammatory phase and a later occlusive phase driven by progressive vascular remodeling [4]. Constitutional symptoms may be subtle or absent. Renovascular hypertension is among the most frequent presentations and reflects involvement of the renal arteries, as illustrated in the present case by complete occlusion of the left renal artery with compensatory contralateral renal hypertrophy.

Hypertension in Takayasu arteritis is multifactorial but most commonly renovascular in origin. Stenosis or occlusion of the renal arteries leads to reduced renal perfusion, triggering activation of the renin-angiotensin-aldosterone system and resulting in vasoconstriction and sodium retention [5]. In addition, decreased aortic compliance due to inflammatory wall thickening and fibrosis contributes to increased vascular stiffness and systolic hypertension. Less frequently, involvement of the aortic arch and its branches may alter baroreceptor function, further contributing to blood pressure dysregulation [6].

Inflammatory biomarkers, including elevated C-reactive protein and erythrocyte sedimentation rate, support systemic inflammation but remain non-specific; hence, imaging is pivotal for diagnosis and disease characterization. Cross-sectional modalities, such as computed tomography angiography, typically demonstrate concentric mural thickening, contrast enhancement, and progressive luminal narrowing or occlusion [7].

The American College of Rheumatology (ACR) classifies Takayasu arteritis using a combination of clinical and angiographic features, requiring at least three criteria among young age at onset, limb claudication, decreased brachial pulse, blood pressure asymmetry, vascular bruit, or characteristic arteriographic abnormalities [7]. The European Alliance of Associations for Rheumatology (EULAR) criteria are centered on mandatory angiographic involvement of the aorta or its major branches associated with clinical or biological evidence, such as pulse deficits, hypertension, vascular bruit, or elevated inflammatory markers [7]. In our young female patient, circumferential outward thickening of the suprarenal aorta, concurrent superior mesenteric artery ostial stenosis, and unilateral renal artery occlusion are highly suggestive of Takayasu vasculitis. Consequently, the diagnosis was supported by young age at presentation, hypertension, elevated inflammatory markers, and characteristic vascular imaging findings consistent with ACR and EULAR classification criteria. The coexistence of complete renal artery occlusion and renal atrophy is consistent with chronic vascular remodeling, whereas mildly elevated inflammatory markers suggest potential ongoing disease activity. Although PET-CT and MRI may provide additional assessment of vascular inflammation, their absence did not modify the therapeutic approach, which was driven by the clinical presentation, laboratory findings, and definitive structural abnormalities on CT angiography indicative of advanced renovascular disease.

Inflammatory arterial disease is also associated with endothelial injury and disturbed flow dynamics, which may predispose to secondary thrombus formation. Although no imaging evidence of intraluminal thrombus was identified in this case, a totally occluded artery in a young patient raises the possibility of thrombus formation. D-dimer is often normal in Takayasu arteritis, particularly in chronic fibrotic disease. Elevation may be observed in cases complicated by acute thrombotic events.

Management is primarily medical, based on systemic corticosteroids and adjunctive immunosuppressive agents in order to control vascular inflammation and prevent disease progression [8]. Optimal antihypertensive therapy is mandatory, particularly in renovascular hypertension, and often requires combination regimens. Blood pressure was successfully controlled to approximately 120/70 mmHg with low-dose amlodipine. Low-dose aspirin was initiated as part of the vascular protective strategy given the presence of severe ostial stenosis and complete renal artery occlusion in a patient with large-vessel vasculitis. Although no imaging of thrombosis was identified and D-dimer levels were normal, antiplatelet therapy was considered appropriate to mitigate potential ischemic and thrombotic risks associated with endothelial injury and critical arterial narrowing in Takayasu arteritis.

Revascularization strategies, including percutaneous transluminal angioplasty and surgical bypass, are reserved for selected cases. Current evidence supports intervention in patients with critical stenoses causing refractory hypertension or progressive renal impairment despite medical therapy [9]. Whenever feasible, procedures should be deferred until disease quiescence to reduce the risk of restenosis and peri-procedural complications [10].

Renal viability assessment using differential renal function studies or nuclear scintigraphy may be considered in selected cases to guide revascularization; however, it was not performed in the present case given the presence of complete left renal artery occlusion with marked renal atrophy (53 mm) on CT imaging, consistent with advanced chronic ischemic nephropathy and presumed irreversible loss of function. In this context, revascularization was not indicated. Moreover, in Takayasu arteritis, long-standing fibrotic lesions are associated with poor procedural outcomes and high restenosis rates, further supporting a conservative therapeutic approach.

Follow-up in Takayasu arteritis is essential, as clinical and biological markers may not reliably reflect disease activity. In this patient with left renal artery occlusion and an atrophic kidney, follow-up imaging is required to monitor disease activity in the remaining renal, mesenteric, and aortic vascular beds. Magnetic resonance angiography is preferred to assess both luminal changes and vessel wall inflammation. Imaging should be performed at 3-6 months after initiation of corticosteroid therapy to evaluate response, then annually if stable [10].

This case is notable for the absence of several classical features of Takayasu arteritis, including limb claudication, pulse deficits, vascular bruits, and blood pressure asymmetry, highlighting the insidious nature of the disease. In young patients, unexplained or severe hypertension associated with elevated inflammatory markers and imaging evidence of large-vessel involvement should raise suspicion for Takayasu arteritis, even in the absence of typical peripheral vascular signs. Early recognition is essential to prevent irreversible vascular and renal damage.

Overall, we underscore the need to consider large-vessel vasculitis in young patients presenting with secondary hypertension and systemic inflammation, and highlight the pivotal role of imaging in guiding both diagnosis and therapeutic decision-making.

Several limitations should be acknowledged. First, assessment of disease activity relied primarily on inflammatory markers and CT angiographic findings, without confirmation by PET-CT or MRI, which were not performed because, in our case, they were unlikely to modify immediate management. Second, renal viability was inferred from severe unilateral renal atrophy and complete renal artery occlusion on imaging, without complementary nuclear functional assessment. Finally, as a single-case report, the findings and therapeutic considerations may not be generalizable to all presentations of Takayasu arteritis.

## Conclusions

Our case describes an incidental diagnosis of advanced multivessel disease in an otherwise asymptomatic young patient presenting with moderate hypertension and established unilateral renal atrophy, illustrating the insidious and clinically silent progression of the disease. It underscores the key role of imaging in diagnosis and in distinguishing active inflammation from irreversible vascular damage, although advanced modalities, such as PET-CT, MRI, or histopathology, were not available. The presence of renal artery occlusion with established atrophy highlights the importance of assessing organ viability. Management should prioritize immunosuppressive therapy and optimal blood pressure control, with revascularization reserved for selected cases with salvageable tissue and inactive disease. Antiplatelet therapy may be considered on an individual basis in view of limited but suggestive observational evidence of reduced ischemic events in large-vessel vasculitis.
